# Uses of Laccases in the Food Industry

**DOI:** 10.4061/2010/918761

**Published:** 2010-09-30

**Authors:** Johann F. Osma, José L. Toca-Herrera, Susana Rodríguez-Couto

**Affiliations:** ^1^Department of Electrical and Electronics Engineering, University of the Andes, Carrera 1 No. 18A-12, Bogota, Colombia; ^2^Department of Nanobiotechnology, University of Natural Resources and Applied Life Sciences (BOKU), Muthgasse 11, 1190 Vienna, Austria; ^3^Unit of Environmental Engineering, CEIT, Paseo Manuel de Lardizábal 15, 20018 San Sebastián, Spain; ^4^IKERBASQUE, Basque Foundation for Science, Alameda Urquijo 36, 48011 Bilbao, Spain

## Abstract

Laccases are an interesting group of multi copper enzymes, which have received much attention of researchers in the last decades due to their ability to oxidise both phenolic and nonphenolic lignin-related compounds as well as highly recalcitrant environmental pollutants. This makes these biocatalysts very useful for their application in several biotechnological processes, including the food industry. Thus, laccases hold great potential as food additives in food and beverage processing. Being energy-saving and biodegradable, laccase-based biocatalysts fit well with the development of highly efficient, sustainable, and eco-friendly industries.

## 1. Introduction

**Figure 1:**
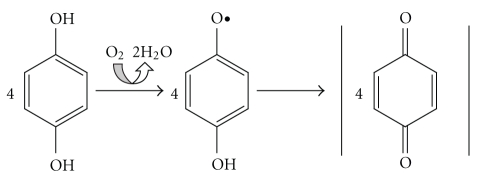
Reactions on phenolic compounds catalysed by laccases (extracted from [10]).

Laccases (*p*-diphenol:dioxygen oxidoreductases; EC 1.10.3.2) are particularly abundant in white-rot fungi, which are the only organisms able to degrade the whole wood components [1]. Fungal laccases are secreted, glycosylated proteins with two disulphide bonds and four copper atoms distributed in one mononuclear termed T1 (where the reducing substrate place is) and one trinuclear cluster T2/T3 (where oxygen binds and is reduced to water) [2]. Thus, electrons are transferred from substrate molecules through the T1 copper to the trinuclear T2/T3 centre. After the transfer of four electrons, the dioxygen in the trinuclear centre is reduced to two molecules of water [3, 4] (Figure 1).

**Figure 2:**
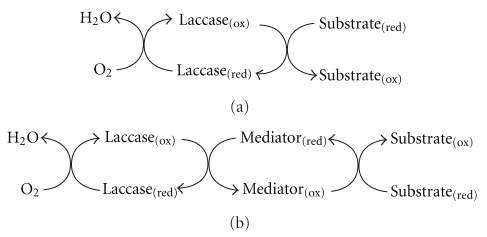
Schematic representation of laccase-catalysed redox cycles for substrates oxidation in the absence (a) or in the presence (b) of redox mediators (extracted from [11], with kind permission of Elsevier Ltd.)

From a mechanistic point of view, the reactions catalysed by laccases can be represented by one of the schemes shown in Figure 2. The simplest case (Figure 2(a)) is the one in which the substrate molecules are oxidised to the corresponding radicals by direct interaction with the copper cluster. Frequently, however, the substrates of interest cannot be oxidised directly by laccases, either because they are too large to penetrate into the enzyme active site or because they have a particularly high redox potential. By mimicking nature, it is possible to overcome this limitation with the addition of so-called “redox mediators”, which are low-weight molecular compounds that act as intermediate substrates for laccases, whose oxidised radical forms are able to interact with the bulky or high redox potential substrate targets (Figure 2(b)).

In nature, the role of laccases is to degrade lignin in order to gain access to the other carbohydrates in wood (cellulose and hemicellulose). Their low substrate specificity allows laccases to degrade compounds with a structure similar to lignin, such as polyaromatic hydrocarbons (PAHs), textile dyes, and other xenobiotic compounds [2]. This together with the simple requirements of laccase catalysis (presence of substrate and O_2_) makes laccases both suitable and attractive for industrial applications.

Typical fungal laccases are extracellular proteins of approximately 60–70 kDa with acidic isoelectric point around pH 4.0 [5]. They are generally glycosylated, with an extent of glycosylation ranging between 10 and 25% and only in a few cases higher than 30% [6, 7]. This feature may contribute to the high stability of the enzyme [8].

A few laccases are at present in the market for textile, food and other industries (Table 1), and more candidates are being actively developed for future commercialisation [9]. A vast amount of industrial applications for laccases have been proposed which include pulp and paper, textile, organic synthesis, environmental, food, pharmaceutical, and nano-biotechnology. Being energy-saving and biodegradable, laccase-based biocatalysts fit well with the development of highly efficient, sustainable, and eco-friendly industries.

This paper reviews the potential application of laccases in the food industry. The utilisation of whole laccase-producing microorganisms is not considered in the present paper.

## 2. Application of Laccases in the Food Industry

Many laccase substrates, such as carbohydrates, unsaturated fatty acids, phenols, and thiol-containing proteins, are important components of various foods and beverages. Their modification by laccase may lead to new functionality, quality improvement, or cost reduction [12, 13].

### 2.1. As Additives in Food and Beverage Processing

Laccases can be applied to certain processes that enhance or modify the colour appearance of food or beverage.

#### 2.1.1. Wine Stabilisation

Wine stabilisation is one of the main applications of laccase in the food industry as alternative to physical-chemical adsorbents [12]. Musts and wines are complex mixtures of different chemical compounds such as ethanol, organic acids (aroma), salts, and phenolic compounds (colour and taste). Polyphenol removal must be selective to avoid an undesirable alteration in the wine's organoleptic characteristics. Laccase presents some important requirements when used for the treatment of polyphenol removal in wines such as stability in acid medium and reversible inhibition with sulphite [14]. Additionally, a laccase has been commercialised for preparing cork stoppers for wine bottles [15]. The enzyme oxidatively reduces the characteristic cork taint and/or astringency, which is frequently imparted to aged bottled wine.

#### 2.1.2. Beer Stabilisation

The storage life of beer depends on different factors such us haze formation, oxygen content, and temperature. The former is produced by small quantities of naturally-occurring proanthocyanidins, polyphenols that generate protein precipitation and, therefore, the formation of haze [16]. This type of complex is commonly found as chill-haze and appears during cooling processes but may re-dissolve at room temperature or above [12]. Even products that are haze-free at the time of packing can develop this type of complex during long-term storage. Thus, the formation of haze has been a persistent problem in the brewing industry [17]. The use of laccases for the oxidation of polyphenols as an alternative to the traditional treatment has been tested by different authors [16, 18, 19]. However, laccases have also been used for the removal of oxygen at the end of the beer production process. According to Mathiasen [16], laccase could be added at the end of the process in order to remove the unwanted oxygen in the finished beer, and thereby the storage life of beer is enhanced. Also, a commercialised laccase preparation named “Flavourstar”, manufactured by Novozymes A/S, is marketed for using in brewing beer to prevent the formation of off-flavour compounds (e.g., trans-2-nonenal) by scavenging the oxygen, which otherwise would react with fatty acids, amino acids, proteins and alcohol to form off-flavour precursors [20] (Table 1).

#### 2.1.3. Fruit Juice Processing

Enzymatic preparations have been studied since the decade of the 1930s for juice clarification [21]. The interaction between proteins and polyphenols results in the formation of haze or sediment in clear fruit juices. Therefore, clear fruit juices are typically stabilised to delay the onset of protein-polyphenol haze formation [22]. Several authors have proposed the use of laccase for the stabilisation of fruit juices [23–30]; however, results are contradictory. On one hand, Sammartino et al. [24] compared the treatment of apple juice with a conventional method (SO_2_ added as metabisulfite, polyvinylpolypyrrolidone (PVPP), bentonite) with the use of free and immobilised laccase. They showed that the enzymatically treated juice was less stable than the one conventionally treated. Also, Giovanelli and Ravasini [25] and Gökmen et al. [31] showed by stability tests of ultrafiltrated samples that laccase treatment increased the susceptibility of browning during storage. On the other hand, Cantarelli [30] used a mutant laccase from *Polyporus versicolor* to treat black grape juice. He showed a removal of 50% of total polyphenols and higher stabilisation than the physical-chemical treatment.

The use of laccase in conjunction with a filtration process has shown better results. Thus, Ritter et al. [27] and Maier et al. [29] obtained a stable and clear apple juice by applying laccase in conjunction with cross-flow-filtration (ultrafiltration) in a continuous process without the addition of finishing agents. Cantarelli and Giovanelli [28] reported that the use of laccase followed by ‘‘active” filtration or ultrafiltration, by the addition of ascorbic acid and sulphites, improved colour and flavour stability in comparison to conventional treatments. Also, Stutz [26] used laccase and ultrafiltration to produce clear and stable juice concentrates with a light colour.

Artik et al. [32] studied the effect of laccase application on clarity stability of sour cherry juice. They found that high clarity was obtained by adding laccase in case of heating to 50°C for 6 h and filtering through 20 kDa membrane after 1 h of oxidation. Also, the phenolic content decreased by around 70%.

More recently, Neifar et al. [23] used a combined laccase-ultrafiltration process for controlling the haze formation and browning of the pomegranate juice. The optimised treatment with laccase (laccase concentration 5 U/mL; incubation time 300 min; incubation temperature 20°C) followed by ultrafiltration led to a clear and stable pomegranate juice.

#### 2.1.4. Baking

Laccases are currently of interest in baking due to their ability to cross-link biopolymers. The use of laccase in baking is reported to result in an increased strength, stability, and reduced stickiness and thereby improved machinability of the dough; in addition, an increased volume and an improved crumb structure and softness of the baked product were observed [33, 34].

Selinheimo et al. [35] showed that a laccase from the white-rot fungus *Trametes hirsuta* increased the maximum resistance of dough and decreased the dough extensibility in both flour and gluten doughs. It was concluded that the effect of laccase was mainly due to the cross-linking of the esterified ferulic acid (FA) on the arabinoxylan (AX) fraction of dough resulting in a strong AX network. Gluten dough treated with laccase also showed some hardening suggesting that laccase can also act to some extent on the gluten protein matrix. The hardening effect of laccase was, however, clearly weaker in gluten dough. Thus, the AX fraction in flour dough is the predominant substrate for laccase, and its activity caused the hardening effect. Interestingly, laccase-treated flour dough softened as a result of prolonged incubation: the extent of softening increasing as a function of laccase dosage. It is proposed that softening phenomenon is due to radical catalysed breakdown of the cross-linked AX network.

Renzetti et al. [36] showed that a commercial laccase preparation significantly improved the bread-making performances of oat flour and the textural quality of oat bread by increasing specific volume and lowering crumb hardness and chewiness. The improved bread-making performances could be related to the increased softness, deformability and elasticity of oat batters with laccase supplementation.

#### 2.1.5. Improving of Food Sensory Parameters

The physico-chemical deterioration of food products is a major problem related to the evolution of storing and distribution systems and influences the consumer's perception of the product quality. Thus, different uses of laccase have promoted odour control, taste enhancement, or reduction of undesired products in several food products.

Takemori et al. [37] used crude laccase from *Coriolus versicolor* to improve the flavour and taste of cacao nib and its products. Bitterness and other unpleasant tastes were removed by the laccase treatment, and the chocolate manufactured from the cacao mass tasted better than the control.

Another type of food products that may use laccase to improve sensory parameters is oil. Oil products may be deoxygenated by adding an effective amount of laccase [38]. Oils, especially vegetable oils (e.g., soybean oil), are present in many food items such us dressings, salads, mayonnaise, and other sauces. Soybean oil contains a large amount of linoleic and linolenic acids that can react with dissolved oxygen in the product producing undesirable volatile compounds. Therefore, the flavour quality of some oils may be improved by eliminating the oxygen present in the oils. Other food products (e.g., juices, soups, concentrates, puree, pastes, and sauces) can also be deoxygenated by the mean of laccase [39].

Bouwens et al. [40, 41] reported that the colour of tea-based products could be enhanced when treated with laccase from a *Pleurotus* species. In the same way, chopped olives in an olive-water mixture were treated with laccase from *Trametes villosa*. In this case, the bitterness was considerably reduced while the colour turned darker compared to the controls (Novo Nordisk A/S, 1995).

Tsuchiya et al. [42] used a recombinant laccase from *Myceliophthora thermophilum* and chlorogenic acid to control the malodour of cysteine. They showed that enzymatically treated cysteine presented a very weak odour while the nontreated cysteine presented a strong characteristic H_2_S odour. HPLC analysis showed the reduction of more than 50% of cysteine.

#### 2.1.6. Sugar Beet Pectin Gelation

The sugar beet pectin is a functional food ingredient that can form thermo-irreversible gels. These types of gels are very interesting for the food industry as can be heated while maintaining the gel structure.

Norsker et al. [43] analysed the gelling effect of two laccases and a peroxidase in food products. They found that laccases were more efficient as gelling agents in luncheon meat and milk than peroxidase. In addition, in many countries it is prohibited to add hydrogen peroxide to food products making it impossible to use peroxidases as gelling agents. Hence, it is more realistic to add laccase to food products.

Kuuva et al. [44] reported that by using laccases as cross-linking agents together with calcium, the ratio of covalent and electrostatic cross-links of sugar beet pectin gels can be varied and it can be possible to tailor different types of gel structures.

Littoz and McClements [45] showed that laccase could be used to covalently cross-link beet pectin molecules adsorbed to the surfaces of protein-coated lipid droplets at pH 4.5, thus suggesting that emulsions with improved functional performance could be prepared using a biomimetic approach that utilised enzymes (laccases) to cross-link adsorbed biopolymers.

### 2.2. Determination of Certain Compounds in Beverages

The use of laccases for improving the sensing parameters of food products is not limited to treatment processes but also to diagnosis systems. In this regard, different amperometric biosensors based on laccases have been developed to measure polyphenols in different food products (e.g., wine, beer, and tea). Thus, Ghindilis et al. [46] showed the practical validity of a biosensor based on immobilised laccase in analysing tannin in tea of different brands.

Montereali et al. [47] reported the detection of polyphenols present in musts and wines from Imola (Italy) through an amperometric biosensor based on the utilisation of tyrosinase and laccase from *Trametes versicolor*. Both enzymes were immobilised on graphite screen-printed electrodes modified with ferrocene. Biosensors exhibited a good sampling behaviour compared to that obtained from spectrophotometric analysis; however, the presence of SO_2_ clearly inhibited the enzymatic activity, and, thus, the measurements on musts and wines recently bottled were seriously affected.

Di Fusco et al. [48] reported the development of an amperometric biosensor based on laccases from *T. versicolor *and* T. hirsuta* for the determination of polyphenol index in wines. Enzymes were immobilised on carbon nanotubes screen-printed electrodes using polyazetidine prepolymer (PAP). They showed that biosensor performance depended on the laccase source. Thus, values obtained by using *T. hirsuta* laccase were close to those determined by Folin-Ciocalteu method whereas polyphenol index measured with *T. versicolor* laccase was discordant to that found with the reference assay.

Prasetyo et al. [49] studied the use of tetramethoxy azobismethylene quinone (TMAMQ) for measuring the antioxidant activity of a wide range of structurally diverse molecules present in food and humans. TMAMQ was generated by the oxidation of syringaldazine with laccases and used to detect the antioxidant activity present in different food products. 

Ibarra-Escutia et al. [50] developed and optimised an amperometric biosensor based on laccase from *T. versicolor* for monitoring the phenolic compounds content in tea infusions. The biosensor developed showed an excellent stability and exhibited good performance in terms of response time, sensitivity, operational stability, and manufacturing process simplicity and can be used for accurate determination of the phenolic content without any pretreatment of the sample.

### 2.3. Bioremediation of Food Industry Wastewater

The presence of phenols in agroindustrial effluents has attracted interest for the application of laccase-based processes in wastewater treatment and bioremediation. The presence of phenolic compounds in drinking and irrigation water or in cultivated land represents a significant health and/or environmental hazard. With government policies on pollution control becoming more and more stringent, industries have been forced to look for more effective treatment technologies for their wastewater.

Some fraction of *beer factory wastewater* represents an important environmental concern due to its high content in polyphenols and dark brown colour.

*Distillery wastewater* is generated during ethanol production from fermentation of sugarcane molasses (vinasses). It produces a serious ecological impact due to its high content in soluble organic matter and its intense dark brown colour. In fact, vinasses represent a major environmental problem for the ethanol production industry and they are considered as the most aggressive by-product generated by sugar-cane factories. Most of the organic matter present in the vinasses can be diminished by conventional anaerobic-aerobic digestion, but the colour is hardly removed by these treatments [51] making this effluent a potential water pollutant blocking out light from rivers and streams thereby preventing oxygenation by photosynthesis and provoking their eutrophication.

Strong and Burgess [52] studied the fungal (*Trametes pubescens*) and enzymatic (laccase from *T. pubescens*) remediation of different distillery wastewater and found that the fungal culture displayed much better properties than laccase alone in removing both the total phenolic compounds and colour. 

*Olive mill wastewater (OMW)* is a characteristic by-product of olive oil production and a major environmental problem in the Mediterranean area. Thus, 30 million m^3^ of OMW is produced in the Mediterranean area [53] which generate 2.5 litres of waste per litre of oil produced [54]. OMW contains large concentrations of phenol compounds (up to 10 g/L) [54, 55], which are highly toxic [52, 56]. Also, it has high chemical and biochemical oxygen demands (COD and BOD, resp.) [57].

OMW is characterised by a colour variable from dark red to black depending on the age and type of olive processed [58], low pH value (~5), high salt content and high organic load with elevated concentrations of aromatic compounds [59], fatty acids, pectins, sugar, tannins and phenolic compounds, in particular polyphenols [58]. The presence of a large number of compounds, many with polluting, phytotoxic, and antimicrobial properties [60], renders OMW a waste with high harmful effects towards humans and environment and makes its disposal one of the main environmental concerns in all producing countries.

Martirani et al. [61] reported that the treatment of an OMW effluent collected at an olive oil factory in Abruzzo (Italy) with a purified laccase from *Pleurotus ostreatus* significantly decreased its phenolic content (up to 90%) but no reduction of its toxicity was observed when tested on *Bacillus cereus*.

Gianfreda et al. [62] showed that laccase from *Cerrena unicolor *was able to oxidise different phenolic substances usually present in OMW with oxidation percentages ranging from 60 to 100% after 24 h of laccase incubation.

D'Annibale et al. [63] used a laccase from the white-rot fungus *Lentinula edodes* immobilised on chitosan to treat OMV from an olive oil mill located in Viterbo (Italy). They found that the treatment of the OMW with immobilised laccase led to a partial decolouration as well as to significant abatements in its content in polyphenols, and orthodiphenols combined with a decreased toxicity of the effluent. They also showed that an oxirane-immobilised laccase from *L. edodes* efficiently removed the OMW phenolics [64].

Casa et al. [65] investigated the potential of a laccase from *L. edodes* in removing OMW phytotoxicity. For this, they performed germinability experiments on durum wheat (*Triticum durum*) in the presence of different dilutions of raw or laccase-treated OMW. The treatment with laccase resulted in a 65% and an 86% reduction in total phenols and orthodiphenols, respectively, due to their polymerisation as revealed by size-exclusion chromatography. In addition, germinability of durum wheat seeds was increased by 57% at a 1 : 8 dilution and by 94% at a 1 : 2 dilution, as compared to the same dilutions using untreated OMW.

Attanasio et al. [66] studied the application of a non-isothermal bioreactor with laccases from *T. versicolor *immobilised on a nylon membrane to detoxify OMW and showed that the technology of non-isothermal bioreactors was very useful in the treatment of OMW.

Jaouani et al. [67] studied the role of a purified laccase from *Pycnoporus coccineus* in the degradation of aromatic compounds in OMW. They found that the treatment of OMW with laccase showed similar results to those reported with the fungus indicating that laccase plays an important role in the degradative process. Berrio et al. [68] studied the treatment of OMW with a laccase from *P. coccineus* immobilised on Eupergit C 250L. Gel filtration profiles of the OMW treated with the immobilised enzyme (for 8 h at room temperature) showed both degradation and polymerisation of the phenolic compounds.

Quaratino et al. [69] reported that phenols were the main determinants for OMW phytotoxicity and showed that the use of a commercial laccase preparation (DeniLite, Novo Nordisk, Denmark) might be very promising for a safer agronomic use of the wastewater.

Iamarino et al. [70] studied the capability of a laccase from* Rhus vernicifera* to degrade and detoxify two OMW samples of different complexity and composition.

Pant and Adholeya [71] used a concentrated enzymatic extract from solid-state fermentation (SSF) cultures of different fungi on wheat straw to decolourise a distillery effluent. They reported a maximum decolouration of 37% in the undiluted distillery effluent using the extract of *Pleurotus florida* EM1303 which was attributed to its high laccase production.

## 3. Future Trends and Perspectives

This paper shows that laccase has a great potential application in several areas of food industry. However, one of the limitations to the large-scale application of laccases is the lack of capacity to produce large volumes of highly active enzyme at an affordable cost (Table 2). The use of inexpensive sources for laccase production is being explored in recent times. In this regard, an emerging field in management of industrial wastewater is exploiting its nutritive potential for production of laccase enzymes. Besides solid wastes, wastewater from the food processing industry is particularly promising for that.

## Figures and Tables

**Table 1:** Commercial preparations based on laccases for industrial processes.

	Main application	Brand name	Manufacturer
Food industry	Brewing	Flavourstar	Advanced Enzyme Technologies Ltd. (India)
	Colour enhancement in tea, etc.	LACCASE Y120	Amano Enzyme USA Co. Ltd.
	Cork modification	Suberase	Novozymes (Denmark)

Paper industry	Pulp bleaching	Lignozym-process	Lignozym GmbH (Germany)
	Paper pulp delignification	Novozym 51003	Novozymes (Denmark)

Textile Industry	Denim bleaching	Bleach Cut 3-S	Season Chemicals (China)
	Denim finishing	Cololacc BB	Colotex Biotechnology Co. Ltd. (Hong Kong)
	Denim bleaching	DeniLite	Novozymes (Denmark)
	Denim finishing	Ecostone LC10	AB Enzymes GmbH (Germany)
	Denim finishing	IndiStar	Genencor Inc. (Rochester, USA)
	Denim finishing	Novoprime Base 268	Novozymes (Denmark)
	Denim bleaching and shading	Primagreen Ecofade LT100	Genencor Inc. (Rochester, USA)
	Denim bleaching	ZyLite	Zytex Pvt. Ltd. (India)

**Table 2:** Some prices of commercially available laccases (extracted from [12], with kind permission of Elsevier Ltd).

	Quantity (Units)^a^	Price
From *Agaricus bisporus *	10.000	305.00 (US$)
	100.000	1.560.00 (US$)
From *Coriolus versicolor *	10.000	250.00 (US$)
	100.000	1.290.00 (US$)

From *Pleurotus ostreatus *	10.000 (concentrate)	150.00 (US$)
	10.000 (purified)	400.00 (US$)
	100.000 (concentrate)	650.00 (US$)
	100.000 (purified)	1,600.00 (US$)

*USBiological*		
(www.usbio.net/)		
From heterologus expression of *Trametes versicolor* laccase in *Saccharomyces cerevisiae *	100 (purified)	169 (US$)

*Sigma-Aldrich*		
From *Rhus vernicfiera *	10,000	72.30 (US$)
From *Agaricus bisporus* (≥1.5 U/mg)	1 g	30.50 (US$)
From *Coriolus versicolor *(≥1 U/mg)	5 g	120.90 (US$)
	1 g	44.00 (US$)
	10 g	358.20 (US$)

*Jena BioScience*		
From *Trametes versicolor*, *Coprinus cinereus* and *Pycnoporus cinnabarinus *	100 U	15.00 (EUR)
	1000 U	75.00 (EUR)

^a^The methodology and expression of laccase activity (Units) are different among the companies.
